# Associations Between Methods of Meeting Sexual Partners and Sexual Practices Among Heterosexuals: Cross-sectional Study in Melbourne, Australia

**DOI:** 10.2196/26202

**Published:** 2021-07-20

**Authors:** Heidi Constantinou, Christopher K Fairley, Jane S Hocking, Catriona S Bradshaw, Edmond P H Choi, Kate Maddaford, Tiffany R Phillips, Eric P F Chow

**Affiliations:** 1 Central Clinical School Monash University Clayton Australia; 2 Melbourne Sexual Health Centre Alfred Health Carlton Australia; 3 Centre for Epidemiology and Biostatistics Melbourne School of Population and Global Health The University of Melbourne Parkville Australia; 4 School of Nursing The University of Hong Kong Hong Kong Hong Kong

**Keywords:** internet, dating apps, mobile phone, sexually transmitted infections, health risk behaviors

## Abstract

**Background:**

The association between meeting partners on the web and sexual practices has been understudied in heterosexuals.

**Objective:**

This study aims to examine the associations between the methods of meeting partners and sexual practices and HIV and sexually transmitted infections (STIs) in heterosexuals.

**Methods:**

We conducted a survey among heterosexuals attending the Melbourne Sexual Health Centre in 2019. This survey asked about the methods through which the participants engaged in meeting their sexual partners, sexual practices, and intravenous drug use (IVDU) over the past 3 months. The participants’ HIV and STI (chlamydia, gonorrhea, and syphilis) status was obtained from clinical testing. Multivariable logistic regression was used to examine the association between each method of meeting and the participants’ sexual practices, IVDU, and STI status.

**Results:**

A total of 698 participants (325 men and 373 women) were included in the study. Most of the participants reported using only one method to meet partners (222/325, 68.3% men; 245/373, 65.7% women; *P*=.05). The men met partners most commonly at social venues (eg, bar, pub, or party; 126/325, 38.8%), whereas the women met partners most commonly through friends or family (178/373, 47.7%). Paying for sex was associated with men meeting partners at sex venues (adjusted odds ratio [AOR] 145.34, 95% CI 26.13-808.51) and on the internet (AOR 10.00, 95% CI 3.61-27.55). There was no association between IVDU and methods of meeting. Social venues were associated with condomless vaginal sex among men (AOR 3.31, 95% CI 1.94-5.71) and women (AOR 2.58, 95% CI 1.61-4.13) and testing positive for STI among men (AOR 3.04, 95% CI 1.24-7.48) and women (AOR 3.75, 95% CI 1.58-8.89).

**Conclusions:**

Heterosexuals who met partners at social venues had a more than threefold risk of testing positive for STIs, indicating that heterosexuals may benefit from health promotion campaigns that are delivered through a public setting.

## Introduction

### Background

There has been an increase in the number of web-based platforms available for individuals to meet sexual partners since the first internet dating site, *Match.com*, was introduced in 1995. The surge in the popularity of social networking sites such as *Instagram,* which was introduced in 2010, led to the launch of other web-based platforms through which individuals could meet partners. The advancing technology of smartphones saw a shift in the nature of web-based dating with the addition of smartphone dating apps, the most popular of which is *Tinder*, which was launched in 2012. As of 2020, *Tinder* had been downloaded 340 million times and claimed to have produced more than 43 billion matches [1].

A total of 2 population-based studies conducted in the United States [2] and Australia [3] have shown that the internet and apps have overtaken the more traditional offline face-to-face methods (eg, through friends or family) for individuals to meet their partners. However, there are limited studies globally that focus on the methods through which heterosexuals meet casual and regular sexual partners. In Australia, gonorrhea and syphilis among heterosexuals have been uncommon since the 1980s, but the incidence of both sexually transmitted infections (STIs) has increased among heterosexuals since the mid-2010s [4,5]. However, the reasons for this rise in the incidence of STIs remain unclear. Although some studies have suggested that meeting partners on the internet or apps are associated with condomless sex [6] and STI acquisition [7,8], other studies did not find this association [9-11]. Thus, the association between web-based meeting methods and sexual risk remains inconclusive. Most of these studies did not stratify by sexual orientation [7,8,10,11]. Very few studies have specifically analyzed heterosexuals exclusively and most were published before 2013 [9], when there were fewer web-based networking platforms (particularly apps). Given that the risk of STIs and sexual practices varies in different sexual orientations and that new web-based networking platforms have continued to surface throughout the mid-2010s to late 2010s [1], it is unclear from these studies whether the same associations of the internet and apps with condom use and STIs can be drawn for heterosexuals in the late 2010s.

### Objective

This study aims to examine the associations between the methods of meeting partners and sexual practices, as well as HIV and STIs, in cisgender heterosexual men and women, which could assist in future HIV and STI prevention and safe sex campaigns.

## Methods

### Study Setting and Population

A cross-sectional study was conducted at the Melbourne Sexual Health Centre (MSHC) in Victoria, Australia, in March and April 2019. The MSHC is a large public sexual health clinic in metropolitan Melbourne. As part of the clinic’s routine care, all new clients who attend the MSHC and clients who have not attended for more than 3 months are asked to complete a questionnaire using computer-assisted self-interview (CASI), which collects information on their sexual activities and demographic characteristics. Following the completion of the CASI, heterosexual clients aged 16 years or older were invited to participate in a voluntary survey on the CASI named *Australian Surveys of Sexual Activities and Practices* (ASAP), which collected additional questions on sexual practice that were not collected as part of the routine CASI questions. Consent was obtained from the participants who selected *yes* on the consent page through the CASI. We defined *heterosexuals* as cisgender male or cisgender female individuals who reported having sex with an opposite-gender partner over the past 12 months and did not report any sexual contact with someone of the same gender over the past 12 months.

The ASAP collected data on the methods through which the participants had met their sexual partners over the past 3 months. The predefined six methods of meeting partners were (1) the internet, (2) apps (eg, *Tinder*), (3) social venues (eg, bar, pub, nightclub, dance, party, disco, and gym), (4) sex venues (eg, sauna, beat, and other sex venues), (5) introduced by friends or family, and (6) other. The participants could choose more than one method. Participants selecting *other* were asked to specify the alternative methods through which they had met their sexual partners. Participants who wrote in *brothel* or *massage parlour* for *other* were recategorized into *sex venues* during analysis.

Data were collected on the participants’ sexual practices and condom use for oral, vaginal, and anal sex; number of regular and casual partners; if they had paid for sex; and intravenous drug use (IVDU) over the past 3 months. All individuals were offered HIV and STI (chlamydia, gonorrhea, and syphilis) testing, and HIV and STI diagnoses were obtained from clinical data on the day the participants completed the survey. HIV and syphilis diagnoses were based on serology. Gonorrhea and chlamydia diagnoses were based on first void urine by nucleic acid amplification test using the Aptima Combo 2 assay (Hologic Panther system; Hologic). No participant tested positive for HIV; therefore, we only analyzed the STI diagnoses.

### Statistical Analysis

Age was categorized into three groups: 16-24 years, 25-34 years, and ≥35 years, as per previous studies [12]. Descriptive statistics, including the frequency and proportion for each method of meeting stratified by age and gender, were calculated. A chi-square test was performed to compare the method of meeting partners between men and women. A chi-square trend test was performed to examine whether there was an increasing or decreasing trend in the method of meeting partners across the three age groups. The *other* category was deemed unreliable because a large proportion of the participants did not specify the alternative methods used to meet partners; hence, we removed *other* from the remaining analyses. Univariable logistic regressions were performed to examine the association between each method of meeting and a range of different variables to determine the sexual risk (eg, the number of sexual partners, condomless sex, having regular and casual partners, testing positive for STI, paying for sex, and IVDU) for men and women separately. Age and the methods of meeting partners were considered potential confounders and adjusted in the multivariable logistic regression analyses. All analyses were performed using SPSS (version 26, IBM Corp). This study was approved by the Alfred Hospital Ethics Committee, Melbourne, Australia (number 571/17).

## Results

### Characteristics of the Study Population

In March and April 2019, there were 2961 heterosexual clients (1506 men and 1455 women) who attended the MSHC and completed the CASI and were invited to participate in the ASAP. Of the 2961 clients, 728 (24.59%) consented and completed the survey, and the proportion who participated did not differ between men (345/1506, 22.91%) and women (383/1455, 26.32%). There was no significant difference in the median ages of the clients who consented versus those who did not consent in both men (28 years vs 29 years; *P*=.16) and women (25 years vs 25 years; *P*=.50). There was also no significant difference in the proportion who consented to participate between Australian-born and overseas-born men (148/345, 25.6% vs 192/345, 21.8%; *P*=.10) and women (111/383, 27.9% vs 262/383, 25.9%; *P*=.50), or STI positivity among men (28/345, 8.1% vs 121/1161, 10.42%; *P*=.21) and women (28/383, 7.3% vs 100/1072, 9.32%); *P*=.41). We excluded 30 participants: 11 reported *other* method only and reported the status of their sexual partner (eg, wife) rather than specifying the *other* method through which they had met, and 19 reported no sexual partners in the past 3 months. Participants who reported the status of their partners under *other*, but had selected an additional method of meeting, were still included in the additional methods of meeting selected but were removed from the *other* category. The remaining 698 participants (325 men and 373 women) were included in the final analysis.

Of the 698 heterosexual participants who were included in the study, the median age was 28 (IQR 24-35) years for men and 25 (IQR 23-29) years for women. Most men and women were born overseas (184/325, 56.6% men and 256/373, 68.6% women). The median total number of partners (including those who only kissed) was 4 (IQR 2-8) for men and 4 (IQR 2-8) for women in the past 3 months. However, the median total number of sexual partners (excluding those who only kissed) was 3 (IQR 2-5) for men and 2 (IQR 2-4) for women.

**Figure 1:**
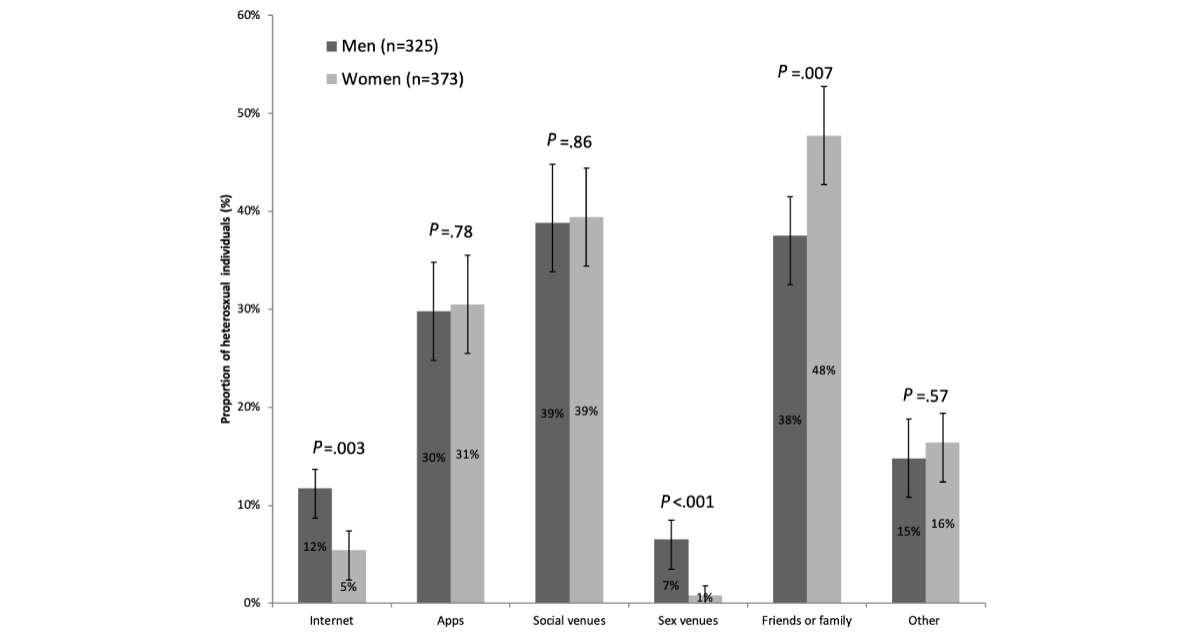
The proportion of heterosexual individuals who engaged in each method of meeting sexual partners over the previous 3 months, stratified by gender. *P* values were calculated from a chi-square test.

Most of the participants reported using only one method to meet sexual partners (222/325, 68.3% among men; 245/373, 65.7% among women; *P*=.46). Overall, most of the men met their partners through social venues (eg, bar, pub, or party; 126/325, 38.8%), whereas most of the women met their partners through friends or family (178/373, 47.7%). Compared with men, fewer women met sexual partners through the internet (38/325, 11.7% vs 20/353, 5.4%; *P*=.003) and sex venues (21/325, 6.5% vs 3/373, 0.8%; *P*<.001), and more women met partners through friends or family (122/325, 37.5% vs 178/373, 47.7%; *P*=.007; Figure 1). There were no significant differences in the proportion of men and women who met partners through apps, social venues, or other methods (Figure 1). Of the 109 participants who reported *other* methods, 65 (59.6%) specified the method, with most meeting partners through work (n=26), travel and backpacking hostels (n=14), education facilities such as school and university (n=5), and public locations (n=5).

**Figure 2:**
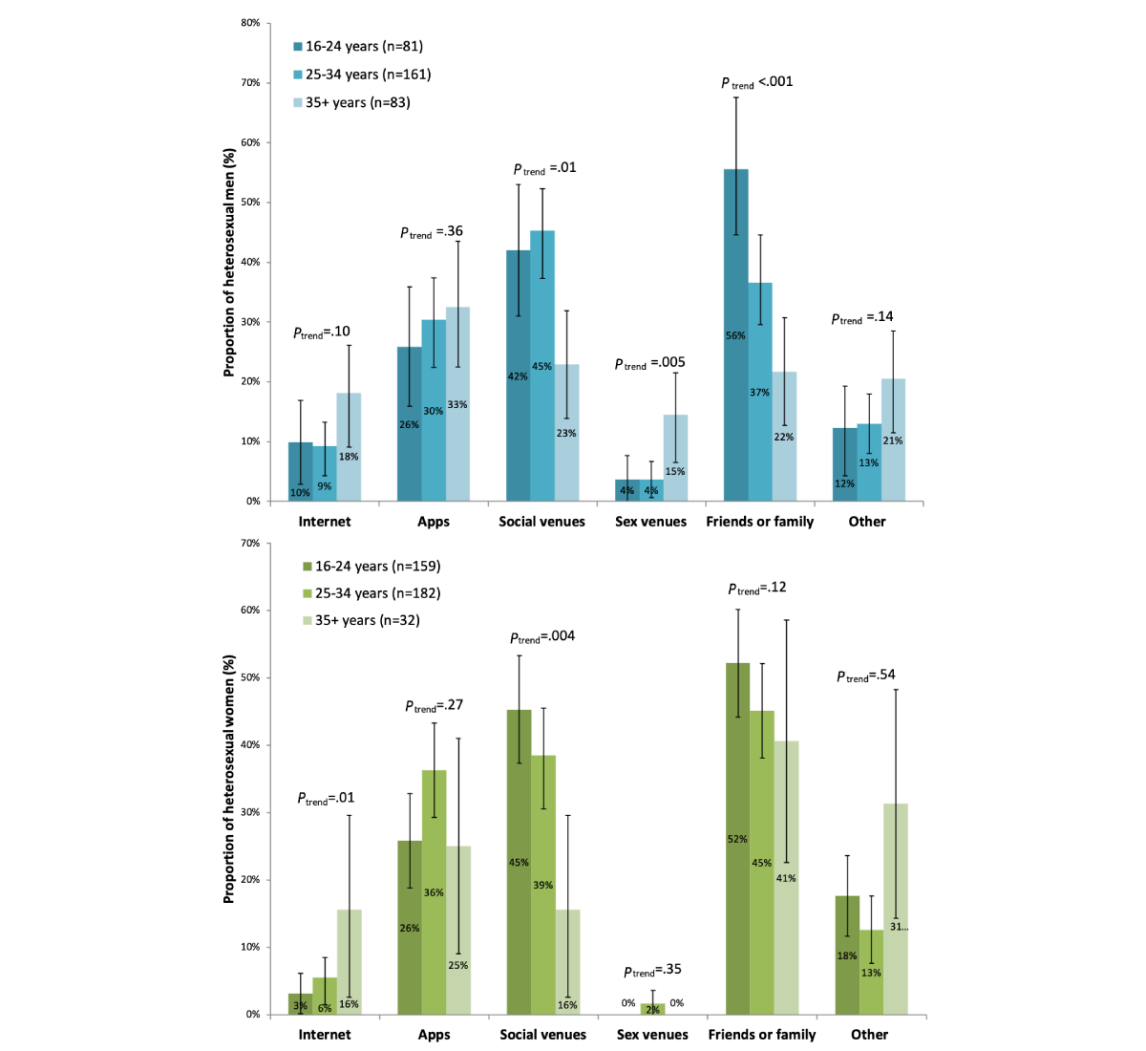
The proportion of heterosexual individuals who engaged in each method of meeting sexual partners over the previous 3 months, stratified by age. *P* values were calculated from a chi-square trend test.

An age pattern was observed for some methods. Among men, the use of sex venues (*P_trend_*=.005) was associated with increasing age, but the use of social venues (*P_trend_*=.01) and friends or family (*P_trend_*<.001) was associated with decreasing age. Among women, the use of the internet (*P_trend_*=.01) was associated with increasing age but the use of social venues (*P_trend_*=.004) was associated with decreasing age (Figure 2).

### Number and Type of Sexual Partners

Men were more likely to report ≥4 total partners, including *only kissing*, if they used social venues (adjusted odds ratio [AOR] 10.93, 95% CI 5.79-20.64) to meet sexual partners, followed by sex venues (AOR 4.16, 95% CI 1.47-11.77), apps (AOR 3.65, 95% CI 1.97-6.77), the internet (AOR 2.70, 95% CI 1.17-6.24), and friends or family (AOR 2.14, 95% CI 1.18-3.88), after adjusting for age and all methods of meeting partners (Table 1). Women were more likely to report ≥4 total partners, including *only kissing*, if they used the internet (AOR 4.97, 95% CI 1.62-15.22) to meet sexual partners, followed by social venues (AOR 4.38, 95% CI 2.64-7.28), apps (AOR 2.53, 95% CI 1.48-4.31), and friends or family (AOR 2.52, 95% CI 1.54-4.14). There was no statistically significant association between meeting at sex venues and the total number of partners among women. The results remained similar for all sexual partners when excluding those who kissed only.

The adjusted odds of having casual partners were highest among men meeting partners at sex venues (AOR 37.84, 95% CI 4.63-309.40), followed by social venues, the internet, apps, and friends or family (Table 1). Accordingly, men were less likely to have regular partners when meeting partners through apps (AOR 0.52, 95% CI 0.31-0.86) and social venues (AOR 0.53, 95% CI 0.33-0.87) but not when meeting them through the internet, friends or family, or at sex venues. The adjusted odds of having casual partners were highest among women meeting partners at social venues (AOR 12.47, 95% CI 5.15-30.20), followed by apps and friends or family but not the internet. All women who met partners at sex venues (N=3) had casual partners, preventing us from performing a logistic regression for sex venues among women. Accordingly, women were less likely to have regular partners when meeting partners through social venues (AOR 0.22, 95% CI 0.13-0.37), followed by apps and friends or family but not through the internet or sex venues.

**Table 1:** Association of method of meeting partners and number and type of sexual partners among heterosexual individuals in the past 3 months.

Sex and method of meeting				Number who had ≥4 partners, including only kissing, n (%)		Adjusted odds ratio (95% CI)^a^		Number who had ≥3 partners, excluding only kissing, n (%)		Adjusted odds ratio (95% CI)^a^		Number who had casual sex partners^b^, n (%)		Adjusted odds ratio (95% CI)^a^		Number who had regular sex partners^c^, n (%)		Adjusted odds ratio (95% CI)^a^
**Men (n=325)**																		
	**Internet**																	
		No (n=287)	164 (57.1)		1		148 (51.6)		1		201 (70)		1		134 (46.7)		1	
		Yes (n=38)	24 (63.2)		2.70 (1.17-6.24)^d^		23 (60.5)		3.27 (1.34-7.95)^d^		32 (84.2)		5.76 (1.98-16.71)^d^		21 (55.3)		1.25 (0.61-2.59)	
	**Apps^e^**																	
		No (n=228)	120 (52.6)		1		103 (45.2)		1		151 (66.2)		1		117 (51.3)		1	
		Yes (n=97)	68 (70.1)		3.65 (1.97-6.77)^d^		68 (70.1)		5.90 (3.09-11.24)^d^		82 (84.5)		5.56 (2.69-11.48)^d^		38 (39.2)		0.52 (0.31-0.86)^d^	
	**Social venues**																	
		No (n=199)	84 (42.2)		1		77 (38.7)		1		121 (60.8)		1		107 (53.8)		1	
		Yes (n=126)	104 (82.5)		10.93 (5.79-20.64)^d^		94 (74.6)		10.92 (5.74-20.78)^d^		112 (88.9)		11.78 (5.62-24.69)^d^		48 (38.1)		0.53 (0.33-0.87)^d^	
	**Sex venues**																	
		No (n=304)	176 (57.9)		1		157 (51.6)		1		213 (70.1)		1		146 (48)		1	
		Yes (n=21)	12 (57.1)		4.16 (1.47-11.77)^d^		14 (66.7)		10.41 (3.47-31.24)^d^		20 (95.2)		37.84 (4.63-309.40)^d^		9 (42.9)		0.42 (0.16-1.12)	
	**Friends or family**																	
		No (n=203)	112 (55.2)		1		99 (48.7)		1		143 (70.4)		1		96 (47.3)		1	
		Yes (n=122)	76 (62.3)		2.14 (1.18-3.88)^d^		72 (59)		3.19 (1.71-5.94)^d^		90 (73.8)		2.91 (1.49-5.71)^d^		59 (48.4)		1.08 (0.66-1.77)	
**Women** **(n=373)**																		
	**Internet**																	
		No (n=353)	195 (55.2)		1		166 (47)		1		289 (81.9)		1		131 (37.1)		1	
		Yes (n=20)	14 (70)		4.97 (1.62-15.22)^d^		13 (65)		5.53 (1.83-16.71)^d^		15 (75)		2.05 (0.39-10.63)		7 (35)		0.44 (0.15-1.31)	
	**Apps^e^**																	
		No (n=258)	136 (52.7)		1		109 (42.2)		1		201 (77.9)		1		107 (41.5)		1	
		Yes (n=115)	73 (63.4)		2.53 (1.48-4.31)^d^		70 (60.9)		3.94 (2.27-6.81)^d^		103 (89.6)		3.51 (1.42-8.69)^d^		31 (27)		0.28 (0.16-0.50)^d^	
	**Social venues**																	
		No (n=226)	103 (45.6)		1		85 (37.6)		1		164 (72.6)		1		106 (46.9)		1	
		Yes (n=147)	106 (72.1)		4.38 (2.64-7.28)^d^		94 (63.9)		4.90 (2.93-8.18)^d^		140 (95.2)		10.38 (3.66-29.43)^d^		32 (21.8)		0.20 (0.12-0.35)^d^	
	**Sex venues**																	
		No (n=370)	207 (55.9)		1		177 (47.8)		1		301 (81.3)		—^f^		137 (37)		1	
		Yes (n=3)	2 (66.7)		2.95 (0.23-38.08)		2 (66.7)		5.54 (0.42-73.39)		3 (100)		—		1 (33.3)		0.32 (0.03-3.91)	
	**Friends or family**																	
		No (n=195)	100 (51.3)		1		84 (43.1)		1		154 (79)		1		80 (41)		1	
		Yes (n=178)	109 (61.2)		2.52 (1.54-4.14)^d^		95 (53.4)		2.90 (1.75-4.80)^d^		304 (82)^g^		3.08 (1.38-6.86)^d^		58 (33)		0.41 (0.24-0.68)^d^	

^a^Odds ratio adjusted according to age and method of meeting.

^b^A total of 35 men declined to answer the question on casual partners in the last 3 months; these participants were added to the *no* group. A total of 29 women declined to answer the question on casual partners in the last 3 months; these participants were added to the *no* group.

^c^A total of 11 men declined to answer the question on regular partners in the last 3 months; these participants were added to the *no* group. A total of 11 women declined to answer the question on regular partners in the last 3 months; these participants were added to the *no* group.

^d^Statistically significant results with *P*<.05.

^e^Mobile dating apps.

^f^All women (N=3) who met partners at sex venues had casual partners, preventing a logistic regression from being performed.

^g^n=373.

### Sexual Practices and Drug Use

Men were more likely to perform oral sex (cunnilingus) with partners they met through apps (AOR 2.62, 95% CI 1.20-5.74) and friends or family (AOR 3.08, 95% CI 1.44-6.58; Table 2). Similarly, men were more likely to receive oral sex (fellatio) from partners met through apps (AOR 7.78, 95% CI 1.75-34.62), social venues (AOR 4.22, 95% CI 1.44-12.34), and friends or family (AOR 3.19, 95% CI 1.13-9.01). Men were more likely to engage in vaginal sex with partners met through friends or family (AOR 6.46, 95% CI 2.11-19.73) and social venues (AOR 2.47, 95% CI 1.05-5.84). Among women, performing fellatio and vaginal sex was not associated with any method of meeting partners (Table 3). Women were more likely to receive cunnilingus from partners met through friends or family (AOR 2.17, 95% CI 1.05-4.46); however, there was no association with any other methods of meeting and receiving cunnilingus. Having anal sex was not associated with any method of meeting partners in either male or female participants.

Men were more likely to have condomless vaginal sex with partners met at social venues (AOR 3.31, 95% CI 1.94-5.71) and were less likely to receive condomless fellatio with partners met at sex venues (AOR 0.09, 95% CI 0.002-0.34; Table 4). Women were more likely to have condomless vaginal sex with partners met at social venues (AOR 2.58, 95% CI 1.61-4.13) and to perform condomless fellatio with partners met through apps (AOR 2.72, 95% CI 1.09-6.77). Neither the internet nor apps were associated with condomless sex among men or women.

The adjusted odds of having paid for sex were highest among men meeting partners at sex venues (AOR 145.34, 95% CI 26.13-808.51), followed by the internet (AOR 10.00, 95% CI 3.61-27.55; Multimedia Appendix 1). There was no association between paying for sex and meeting partners through apps, social venues, or friends or family among men. Only 1 woman paid for sex, and she met the partner through friends or family. There was no association between any methods of meeting partners and IVDU among men and women.

**Table 2:** Association of method of meeting partners and sexual practices among heterosexual men in the past 3 months (N=325).

Method of meeting		Men who performed oral sex (cunnilingus), n (%)	Adjusted odds ratio (95% CI)^a^	Men who received oral sex (fellatio), n (%)	Adjusted odds ratio (95% CI)^a^	Men who had vaginal sex, n (%)	Adjusted odds ratio (95% CI)^a^	Men who had anal sex, n (%)	Adjusted odds ratio (95% CI)^a^
**Internet**									
	No (n=287)	238 (82.9)	1	262 (91.3)	1	257 (89.5)	1	57 (19.9)	1
	Yes (n=38)	30 (78.9)	1.08 (0.43-2.68)	34 (89.5)	1.48 (0.44-4.93)	27 (71.1)	0.47 (0.20-1.13)	11 (28.9)	1.81 (0.81-4.02)
**Apps^b^**									
	No (n=228)	180 (78.9)	1	201 (88.2)	1	195 (85.5)	1	43 (18.9)	1
	Yes (n=97)	88 (90.7)	2.62 (1.20-5.74)^c^	95 (97.9)	7.78 (1.75-34.62)^c^	89 (91.8)	2.14 (0.90-5.09)	25 (25.8)	1.52 (0.85-2.73)
**Social venues**									
	No (n=199)	161 (80.9)	1	175 (87.9)	1	167 (83.9)	1	38 (19.1)	1
	Yes (n=126)	107 (84.9)	1.41 (0.71-2.77)	121 (96)	4.22 (1.44-12.34)^c^	117 (92.9)	2.47 (1.05-5.84)^c^	30 (23.8)	1.66 (0.93-2.99)
**Sex venues**									
	No (n=304)	254 (83.6)	1	279 (91.8)	1	269 (88.5)	1	63 (20.7)	1
	Yes (n=21)	14 (66.7)	0.77 (0.27-2.18)	17 (81)	1.21 (0.34-4.29)	15 (71.4)	0.83 (0.27-2.53)	5 (23.8)	1.55 (0.50-4.77)
**Friends or family**									
	No (n=203)	158 (77.8)	1	180 (88.7)	1	116 (57.1)	1	41 (20.2)	1
	Yes (n=122)	110 (90.2)	3.08 (1.44-6.58)^c^	116 (95.1)	3.19 (1.13-9.01)^c^	118 (96.7)	6.46 (2.11-19.73)^c^	27 (22.1)	1.51 (0.83-2.73)

^a^Odds ratio adjusted according to age and method of meeting.

^b^Mobile dating apps.

^c^Statistically significant results with *P*<.05.

**Table 3:** Association of method of meeting partners and sexual practices among heterosexual women in the past 3 months (N=373).

Method of meeting		Number of women who performed oral sex (fellatio), n (%)	Adjusted odds ratio (95% CI)^a^	Number of women who received oral sex (cunnilingus), n (%)	Adjusted odds ratio (95% CI)^a^	Number of women who had vaginal sex, n (%)	Adjusted odds ratio (95% CI)^a^	Number of women who had anal sex, n (%)	Adjusted odds ratio (95% CI)^a^
**Internet**									
	No (n=353)	329 (93.2)	1	315 (89.2)	1	330 (93.5)	1	60 (17)	1
	Yes (n=20)	19 (95)	1.60 (0.17-15.20)	16 (80)	0.69 (0.20-2.38)	18 (90)	0.82 (0.16-4.22)	7 (35)	2.51 (0.89-7.06)
**Apps^b^**									
	No (n=258)	237 (91.9)	1	226 (87.6)	1	241 (93.4)	1	46 (17.8)	1
	Yes (n=115)	111 (96.5)	2.62 (0.84-8.18)	105 (91.3)	1.90 (0.86-4.20)	107 (93)	1.01 (0.40-2.54)	21 (18.3)	1.12 (0.62-2.04)
**Social venues**									
	No (n=226)	213 (94.2)	1	199 (88)	1	208 (92)	1	34 (19)	1
	Yes (n=147)	135 (91.8)	0.78 (0.32-1.87)	132 (89.8)	1.32 (0.64-2.73)	140 (95.2)	1.61 (0.63-4.13)	24 (16.3)	1.03 (0.58-1.84)
**Sex venues**									
	No (n=370)	346 (93.5)	1	328 (88.6)	—^c^	346 (93.5)	1	65 (17.6)	1
	Yes (n=3)	2 (66.7)	0.19 (0.02-2.58)	3 (100)	—	2 (66.7)	0.21 (0.02-2.71)	2 (66.7)	8.16 (0.67-99.20)
**Friends or family**									
	No (n=195)	181 (92.8)	1	167 (85.6)	1	182 (93.3)	1	32 (16.4)	1
	Yes (n=178)	167 (93.8)	1.30 (0.54-3.12)	164 (92.1)	2.17 (1.05-4.46)^d^	166 (93.3)	0.98 (0.41-2.34)	35 (19.7)	1.45 (0.82-2.56)

^a^Odds ratio adjusted according to age and method of meeting.

^b^Mobile dating apps.

^c^All women (n=3) who met partners at sex venues received cunnilingus, preventing a logistic regression from being performed.

^d^Statistically significant results with *P*<.05.

**Table 4:** Association of method of meeting partners and condomless sex in the past 3 months and sexually transmitted infection status among heterosexual individuals.

Sex and method of meeting				Individuals who received condomless oral sex (fellatio)^a^				Adjusted odds ratio (95% CI)^b^		Individuals who had condomless vaginal sex^a^						Adjusted odds ratio (95% CI)^b^				Individuals who had condomless anal sex^a^						Adjusted odds ratio (95% CI)^b^			Individuals who tested positive for STI^c,d^					Adjusted odds ratio (95% CI)^b^	
				Total, N		Participant, n (%)				Total, N			Participant, n (%)							Total, N			Participant, n (%)						Total, N			Participant, n (%)			
**Men (N=325)**																																			
	**Internet**																																		
		No	262		247 (94.3)		1		257			144 (56)			1				57			38 (66.7)			1			287			25 (8.7)		1		
		Yes	34		31 (91.2)		0.90 (0.20-4.04)		27			15 (55.6)			1.23 (0.52-2.86)				11			5 (45.5)			0.52 (0.11-2.40)			38			1 (2.6)		0.38 (0.05-3.08)		
	**Apps^e^**																																		
		No	201		186 (92.5)		1		195			112 (57.4)			1				43			27 (62.8)			1			228			19 (8.3)		1		
		Yes	95		92 (96.8)		1.68 (0.44-6.41)		89			47 (52.8)			0.93 (0.54-1.59)				25			16 (64)			0.91 (0.29-2.86)			97			7 (7.2)		1.01 (0.39-2.63)		
	**Social venues**																																		
		No	175		160 (91.4)		1		167			76 (45.5)			1				38			23 (60.5)			1			199			10 (5)		1		
		Yes	121		118 (97.5)		2.51 (0.63-10.00)		117			83 (70.9)			3.31 (1.94-5.71)^f^				30			20 (66.7)			0.88 (0.28-2.73)			126			16 (12.7)		3.04 (1.24-7.48)^f^		
	**Sex venues**																																		
		No	279		268 (96.1)		1		269			151 (56.1)			1				63			42 (66.7)			1			304			24 (7.9)		1		
		Yes	17		10 (58.8)		0.09 (002-0.34)^f^		15			8 (53.3)			1.42 (0.47-4.31)				5			1 (20)			0.12 (0.01-1.55)			21			2 (9.5)		2.80 (0.50-15.80)		
	**Friends or family**																																		
		No	180		167 (92.8)		1		166			90 (54.2)			1				41			27 (65.9)			1			203			13 (6.4)		1		
		Yes	116		111 (95.7)		1.25 (0.34-4.55)		118			69 (58.5)			1.50 (0.88-2.56)				27			16 (59.3)			0.62 (0.20-1.93)			122			13 (10.7)		1.53 (0.63-3.74)		
**Women (N=373)**																																			
	**Internet**																																		
		No	329		304 (92.4)		1				330			179 (54.2)			1	60			45 (75)			—^g^			353			26 (7.4)					1
		Yes	19		18 (94.7)		2.56 (0.30-21.71)				18			11 (61.1)			2.03 (0.69-5.92)	7			6 (85.7)			—			20			2 (10)					3.81 (0.71-20.49)
	**Apps^e^**																																		
		No	237		218 (92)		1				241			128 (53.1)			1	46			34 (73.9)			1			258			21 (8.1)					1
		Yes	111		104 (93.7)		1.72 (0.66-4.51)				107			62 (57.9)			1.37 (0.84-2.25)	21			17 (81)			1.14 (0.30-4.37)			115			7 (6.1)					0.83 (0.33-2.10)
	**Social venues**																																		
		No	213		195 (91.5)		1				208			97 (46.6)			1	43			32 (74.4)			1			226			9 (4)					1
		Yes	135		127 (94.1)		1.80 (0.72-4.51)				140			93 (66.4)			2.58 (1.61-4.13)^f^	24			19 (79.2)			1.11 (0.29-4.29)			147			19 (12.9)					3.75 (1.58-8.89)^f^
	**Sex venues**																																		
		No	346		320 (92.5)		—^g^				346			190 (54.9)			—^h^	65			51 (78.5)			—^i^			370			28 (7.6)					—^j^
		Yes	2		2 (100)		—^g^				2			0 (0)			—^h^	2			0 (0)			—^i^			3			0 (0)					—^j^
	**Friends or family**																																		
		No	181		163 (90.1)		1				182			100 (54.9)			1	32			26 (81.3)			1			195			12 (6.2)					1
		Yes	167		159 (95.2)		2.72 (1.09-6.77)^e^				166			90 (54.2)			1.25 (0.79-1.99)	35			25 (71.4)			0.62 (0.16-2.42)			178			16 (9)					1.83 (0.81-4.13)

^a^Only participants who engaged in each sexual activity were included in the analyses of condomless sex.

^b^Odds ratio adjusted according to age and method of meeting.

^c^All women (n=3) who met partners at sex venues tested negative for sexually transmitted infection, preventing a logistic regression from being performed.

^d^STI: sexually transmitted infection (chlamydia, gonorrhea, and syphilis).

^e^Mobile dating apps.

^f^Statistically significant results with *P*<.05.

^g^The logistic regression for women who had condomless anal sex produced an adjusted odds ratio >300 million and did not produce a 95% CI upper limit, preventing reliable interpretation of these results.

^h^All women (n=2) who met partners at sex venues who performed fellatio used a condom, preventing a logistic regression from being performed.

^i^All women (n=2) who met partners at sex venues who had vaginal sex used a condom, preventing a logistic regression from being performed.

^j^All women (n=2) who met partners at sex venues who had anal sex used a condom, preventing a logistic regression from being performed.

### STI Positivity

The STI positivity for men was 8.0% (26/325) and that for women was 7.5% (28/373). There was no association between STI positivity and methods, except for social venues. Both men (AOR 3.04, 95% CI 1.24-7.48) and women (AOR 3.75, 95% CI 1.58-8.89) who met partners at social venues were three times more likely to have an STI (Table 4).

## Discussion

### Principal Findings

Despite the increasing use of web-based networking platforms (eg, the internet or apps) [1], this study shows that face-to-face methods (eg, social venues and friends or family) continue to be the most common methods used among heterosexuals to meet sexual partners. After adjusting for the five different methods of meeting, those who met at sex venues were less likely to have condomless sex, whereas meeting at social venues (eg, bar, pub, or party) was strongly associated with having more sexual partners, condomless sex, and testing positive for an STI. To our knowledge, this is the first study to analyze a range of both long-established and contemporary methods of meeting sexual partners among heterosexuals and their associations with sexual practices and STI diagnoses, providing important insights for future health promotion campaigns. The inconsistencies in our findings compared with those in previous studies are likely due to several reasons. First, our study looked at how heterosexuals met their regular and/or casual partners; however, the American study [2] only examined how heterosexual *couples* met, and casual partners were not considered. Second, our study was conducted exclusively among heterosexuals, whereas the Australian Talks National Survey [3] was conducted among 54,000 Australians from all sexual orientations, which found that apps are the most popular method for Australians to meet partners. This is consistent with a previous Melbourne-based study published in 2016 showing that apps are the most popular method to meet partners among 1902 men who have sex with men (MSM) [13]. These are important distinctions to be aware of if we want to develop health promotion campaigns that target heterosexuals with casual partners, and an indication that current campaigns using web-based platforms may not be reaching most of the heterosexual population. To the best of our knowledge, there have been no studies investigating why heterosexuals are more likely to meet partners through friends or family and why friends or family is associated with performing condomless fellatio among women. Further qualitative research could explore whether individuals feel more comfortable and safe engaging in sexual activities with those they have mutual contacts with.

Our results found that individuals meeting partners at social venues had higher odds of having ≥4 partners among both men and women. In addition, meeting partners at social venues was strongly associated with risks, including condomless vaginal sex and STI positivity in men and women. Social venues such as bars, pubs, nightclubs, and parties are common locations where binge drinking and recreational drug use occur. Individuals who binge drink at social venues are six times more likely to engage in sexual activities [14], providing a likely explanation for social venues, alongside friends or family, being the most common method through which heterosexuals meet sexual partners. Alcohol consumption and recreational drug use at social venues are also strongly associated with engaging in risky sexual behaviors—such as condomless sex—and STIs [14-17], providing a likely explanation for our results. Some studies found an association between STIs and meeting partners through the internet and apps [7,8]; however, these included participants of all sexual orientations and found that the internet and apps were more commonly used by participants identifying as homosexual or bisexual, making it unclear if the same conclusion can be drawn for heterosexuals. Furthermore, the fact that our study analyzed a population of participants attending a sexual health clinic, who are presumably more sexually active than the general population, and still did not find an association with STIs and meeting partners through methods other than social venues strongly supports the notion that meeting on the internet and apps does not increase the risk of heterosexuals testing positive for STIs in the wider community.

Certain types of sex venues such as brothels and massage parlors have been long-standing methods through which individuals meet sex workers whom they pay for sex. We found that men who met partners at sex venues were less likely to receive condomless fellatio. Similarly, condomless vaginal and anal sex were not associated with meeting partners at sex venues. A previous Melbourne study found that consistent condom use was high among 106 female sex workers operating in sex venues such as brothels—90% (95) for fellatio, 98% (104) for vaginal sex, and 100% (106) for anal sex among female sex workers with their male clients—[18] because condoms must be used during sex work in accordance with the law in Victoria [19]. As web-based technology has evolved, the internet has become an increasingly common platform in the sex work industry [20]. This may explain why heterosexual men who met partners on the internet were 10 times more likely to report paying for sex in our study. Almost 40% (15/38) of the men in our study who met partners on the internet had paid for sex, of which one-third reported that they used the internet as their sole method of meeting partners.

Our study includes some limitations. First, this study was conducted at a sexual health clinic, which may not be representative of all heterosexuals in Australia. This is because individuals attending a sexual health clinic may be more sexually active and more likely to have casual partners. Second, we predefined six methods of meeting partners from another survey [13], which was originally designed for MSM. Interpretation of these methods could have varied from participant to participant. The examples of sex venues supplied were more applicable to MSM, and additional methods that we did not list may have been underrepresented because the participants did not specify these alternative methods in the *other* category. Third, our response rate was low (728/2961, 24.58%) among both men and women. It is possible that there are some differences in sexual risk between those who participated and those who did not, although there was no difference in demographic characteristics. Fourth, we did not provide examples of who would classify as a *regular* versus *casual* partner. Previous studies have shown ambiguity around how to classify *fuckbuddies* among MSM [21], and although no such research has been conducted among heterosexuals, there may be a similar conundrum of how to classify certain partners (eg, *friends with benefits*). Further research is needed in this area. Finally, this cross-sectional study can only describe associations between the methods of meeting with sexual practices and outcomes, and we cannot rule out all confounders that may have influenced the results, such as marital status, ethnicity, alcohol use, or recreational drug use [14-17].

### Conclusions

Heterosexuals who met partners at social venues such as bars and nightclubs were more likely to have condomless vaginal sex and had a more than three-fold risk of testing positive for STIs. Most sexual health promotion campaigns are directed toward MSM, who, in contrast, have been shown to meet more sexual partners through apps [13]. Our study indicates that heterosexuals may benefit from more targeted health promotion campaigns that are delivered through a more public setting (eg, advertisements at social venues or physical face-to-face interventions). More research is warranted that further examines the association of different methods of meeting partners with STIs and investigates other potential reasons for the rise in the incidence of STIs.

## Appendix

Multimedia Appendix 1 Association of method of meeting partners and paying for sex and intravenous drug use status among heterosexual individuals.

## Abbreviations

AOR: adjusted odds ratio
ASAP: Australian Surveys of Sexual Activities and Practices
CASI: computer-assisted self-interview
IVDU: intravenous drug use
MSHC: Melbourne Sexual Health Centre
MSM: men who have sex with men
NHMRC: Australian National Health and Medical Research Council
STI: sexually transmitted infection
