# Supplementary material for: Associations Between Methods of Meeting Sexual Partners and Sexual Practices Among Heterosexuals: Cross-sectional Study in Melbourne, Australia
Source: JMIR Form Res. 2021 Jul 20;5(7):e26202. doi: 10.2196/26202 (PMC8335617; doi:10.2196/26202)
Supplement: Multimedia Appendix 1 [file formative_v5i7e26202_app1.docx]

**Supplementary Table 1**: Association of Method of Meeting Partners and Paying for Sex and IVDU^a^ Status Among Heterosexual Individuals

|  | ***Males (N=325)*** | | | | |
| --- | --- | --- | --- | --- | --- |
|  | *Number of Males who paid for sex* | Adjusted OR (95%CI)^b^ | *Number of Males who engaged in IVDU^a^* | Adjusted OR (95%CI)^b^ | |
| *Internet* |  |  |  |  | |
| *No* | 27/287 (9.4%) | 1 | 4/287 (1%) | 1 | |
| *Yes* | 15/38 (40%) | **9.98 (3.61-27.55)**^c^ | 1/38 (3%) | 2.65 (0.23-30.45) | |
| *Apps^d^* |  |  |  |  | |
| *No* | 34/228 (15%) |  | 3/228 (1%) |  | |
| *Yes* | 8/97 (8%) | 0.81 (0.30-2.17) | 2/97 (2%) | 1.57 (0.24-10.52) | |
| *Social-venues* |  |  |  |  | |
| *No* | 32/199 (16%) | 1 | 1/199 (1%) | 1 | |
| *Yes* | 10/126 (8%) | 1.73 (0.63-4.73) | 4/126 (3%) | 8.97 (0.85-94.43) | |
| *Sex-venues* |  |  |  |  | |
| *No* | 23/304 (8%) | 1 | 5/304 (2%) | –^e^ | |
| *Yes* | 19/21 (91%) | **145.34 (26.13-808.51)**^c^ | 0/21 (0%) |  |  |
| *Friends/*  *Family* |  |  |  |  | |
| *No* | 38/203 (19%) | 1 | 4/203 (2%) | 1 | |
| *Yes* | 4/122 (3%) | 0.39 (0.12-1.27) | 1/122 (1%) | 0.49 (0.05-4.75) | |
|  | ***Females (N=373)^f^*** | | | | |
|  | *Number of Females who engaged in IVDU^a^* | | Adjusted OR (95%CI)^b^ | | |
|  |  |  |  | |  |
| *Internet* |  |  |  | |  |
| *No* | 3/348 (1%) | | –^g^ | | |
| *Yes* | 0/19 (0%) | |  |  |  |
| *Apps^e^* |  |  |  | |  |
| *No* | 2/253 (1%) | | 1 | | |
| *Yes* | 1/114 (1%) | | 0.88 (0.15-5.09) | | |
| *Social-venues* |  |  |  | |  |
| *No* | 2/224 (1%) | | 1 | | |
| *Yes* | 1/143 (1%) | | 1.52 (0.28-8.23) | | |
| *Sex-venues* |  |  |  | |  |
| *No* | 3/364 (1%) | | –^h^ | | |
| *Yes* | 0/3 (0%) | |  |  |  |
| *Friends/*  *Family* |  |  |  | |  |
| *No* | 2/190 (1%) | | 1 | | |
| *Yes* | 1/177 (1%) | | 0.40 (0.07-2.19) | | |
|  |  | | | | |

^a^Intravenous Drug Use; ^b^Odds ratio adjusted according to age and method of meeting; ^c^Statistically significant results with p<0.05; ^d^Mobile dating applications; ^e^No males who met through sex-venues engaged in IVDU, preventing a logistic regression from being performed; ^f^Only one female paid for sex, who met their partners through Friends/Family, preventing a logistic regression from being performed; ^g^No females who met through the internet engaged in IVDU, preventing a logistic regression from being performed; ^h^No females who met through sex-venues engaged in IVDU, preventing a logistic regression from being performed.
